# A Longitudinal Study of a Chinese Man Presenting with Non-Fluent/Agrammatic Variant of Primary Progressive Aphasia

**DOI:** 10.3389/fneur.2018.00075

**Published:** 2018-02-16

**Authors:** Xiaoyan Liu, Fangping He, Zhongqin Chen, Ping Liu, Guoping Peng

**Affiliations:** ^1^Department of Neurology, First Affiliated Hospital, Zhejiang University School of Medicine, Hangzhou, China

**Keywords:** Alzheimer’s disease, frontotemporal lobar degeneration, longitudinal assessment, non-fluent, primary progressive aphasia

## Abstract

Primary progressive aphasia (PPA) is a neurodegenerative disease characterized by declining language ability. However, the difficulty in defining the central clinical features in its earliest stage and establishing the dynamics of its progression has led to controversy. We report a 71-year-old man with Han language suffering from non-fluent/agrammatic variant of PPA but presenting as typical Alzheimer’s disease (AD) and confused with logopenic variant of PPA in its early stage, longitudinally describing his clinical characteristics, neuroanatomical basis, and genetic associations, and exploring the underlying pathology. This case highlights a longitudinal data for reliably discriminating among AD and PPA variants and helps to deepen our understanding of Han language non-fluent/agrammatic variant of PPA.

## Introduction

Research advances have enabled the elevated understanding of the clinical characteristics and pathogenesis of such progressive neurodegenerative diseases as Alzheimer’s disease (AD) (1) and primary progressive aphasia (PPA) (2). The central feature of the former is declining memory ability and that of the latter is speech deficit. However, difficulties in defining the initial or primary clinical feature, and heterogeneity as well as overlap existing in these diseases have led to controversy and misdiagnosis, especially in the early stage of disease. In addition, the dynamics of its progression in PPA has not been fully described.

## Patient Consent

Written informed consent was obtained from the guardian of the patient for publication of this case report and any accompanying images.

## Description of the Case

The case of the individual reported here, a Chinese right-handed man with 13 years of education, had been working as an engineer since he was 28 years old and retired at home at age 63. He began to complain of memory loss at age 71, on the other hand, and was found speaking aloud, especially at the moment of making a telephone call, sometimes seemed to speak with a slight lisp, but had fluent speech under other circumstances. His speech deficit was initially ignored. Meanwhile, the cranial nerve, motor and sensory system, and reflexes were intact. Both coordination and gait were unremarkable. In addition, screening tests were performed at this point, but no significant abnormalities were detected in blood tests such as routine blood examination, biochemistry, coagulation function, tumor marker, folate, vitamin B12, and thyroid function, as well as screenings for HIV, syphilis, and hepatitis. Routine CSF analysis, CSF biochemistry, CSF pressure, electroencephalogram, and cerebral artery MRI were normal. Mild cortical atrophy without significant laterality was demonstrated in a brain MRI. Thus, AD was considered and he began to receive treatment of cholinesterase inhibitor discontinuously.

Nevertheless, with the progression of disease, his performance worsened in the domain of memory, so that he was tend to repeatedly do something. Furthermore, he produced fewer and fewer words per minute in the next 2 years, his spontaneous speech was on the decrease to the point that it mainly consisted of short phrases, and had difficulty in speech volume control. Besides, his notes were chaotic, only with the title written down but without detailed contents, or the written sentence not well organized or not coherent; however, his font size and shape was normal and without misspelling. Diminished memory and prominent speech difficulty were further evidenced by neurologic examination and a thorough neuropsychological and speech assessment (3–5) at age 73. In particular, he had a score on the Mini-Mental State Examination (MMSE) of 26/30, Montreal Cognitive Assessment (MoCA) of 23/30, Clock-Drawing Test (CDT) of 4/4, immediate recall, delayed recall, and recognition of auditory verbal learning test of 5/15, 4/15, and 10/15, respectively, Trail Making Test-A and -B full score of 0'47"and 1'18", respectively, and buccofacial apraxia and speech apraxia tests score of normal value. Furthermore, modified Aphasia Battery of Chinese test for language evaluating showed that spontaneous speech was deficient, effortful, and non-fluent, halting speech with sound errors, prosody distortion and grammatical mistakes, sentence repetition, and comprehension, and spontaneous writing was impaired, but word repetition, naming, auditory comprehension, reading, word comprehension, transcribing, and dictating were relatively preserved (Table 1). In addition, an investigation of phonological loop functions (6) indicated impairment in digit, letter, and word span tasks with an effect of word length, but without normal phonological similarity effect (Table 2). Therefore, his central features included aphasia and memory impairment. Apart from AD, PPA was taken into consideration.

**Table 1:** Results of longitudinal language evaluating at age 73 and 75.

Item	First evaluation score	Second evaluation score
Spoken speech		
Spontaneous speech		
Information content	3/5	2/5
Fluency	14/30	12/30
Repetition	92/100	51/100
Naming total	70/82	63/82
Objects/pictures naming	40/40	33/40
Serial naming	8/20	10/20
Color naming	12/12	10/12
Responsive naming	10/10	10/10
Comprehension total	196/230	156/230
Yes/no	56/60	32/60
Auditory word/picture matching	90/90	72/90
Following directions	80/80	52/80
Reading total	110/120	68/120
Oral reading	10/10	8/10
Auditory word/print matching	10/10	7/10
Oral reading and word/picture matching	40/40	18/40
Reading and following written directions	30/30	17/30
Reading and filling up the blanks	20/30	18/30
Writing total	76/79	54/79
Spontaneous writing	32/35	16/35
Writing name and address	10/10	6/10
Written naming of pictures	20/20	10/20
Writing state of illness	2/5	0/5
Writing to dictation	34/34	29/34
Copying	10/10	9/10

**Table 2:** Investigation of phonological loop functions.

	Verbal response		Pointing	
	Digits		Digits	
Sequence length	Auditory	Visual	Auditory	
1	1	1	1	
2	1	0.8	1	
3	1	0.8	1	
4	1	0.8	1	
5	0.6	0.4	0.6	

	**Immediate verbal recall**		**Immediate verbal recall**	
	**Letters similar**		**Letters dissimilar**	
	**Auditory**	**Visual**	**Auditory**	**Visual**

1	0.8	1	1	0.9
2	0.6	0.9	0.9	0.9
3	0.4	0.5	0.5	0.3
	
	**Two words**		**Four words**	
	**Auditory**	**Visual**	**Auditory**	**Visual**

1	1	1	1	1
2	1	1	0.8	0.7
3	0.5	0.3	0.2	0

From the perspective of disease progression of his first 2-year disease course, his core deficit was exclusively aphasia, which supported the diagnosis of PPA. To support our hypothesis, typical AD should be eliminated; meanwhile, PPA subtype should be determined. Importantly, the longitudinal course of this patient was documented, which revealed progressive decline in language functioning along with other cognitive function and activity of daily living. In this regard, at age 74, his speech was non-fluent to the extent that it consisted of one-by-one words, showing difficulty in the comprehension of grammatically complex sentences, but not simple sentences. As a result, he was reluctant to read paper and incapable of taking notes. At age 75, his language functioning worsened to the point that his verbal communication was less and less and non-verbal expression was on the increase. “If possible, he would like to get out of verbal communication,” as his wife said. But he was able to listen and repeat, trace, draw clock, and transcribe. Moreover, he deteriorated markedly in memory and nearly got lost twice, and the disorder of personality was noted while he was unsympathetic, impulsive, and vulnerable and attached little importance to instrumentation. Besides, sphincteric disturbance began to occur. At age 76, his spontaneous speech was usually characterized with residual utterances of one word; such symptoms like illusion and gait disturbance began to present, being a little stooped and tend to fall down, and sphincteric disturbance further worsened. At age 77, his spontaneous speech was no more than utterance of a single word “hao”; he always came up with the feeling that his son was going to visit him and thus he went to open the door again and again. On the other hand, he was limited to wheelchair conveyance little by little on account of subsequent walking disturbance and could not take care of himself at all. Besides, he gradually acted as a child and enjoyed embrace from his family. Finally, he died of aspiration pneumonia at this age. To calculate the extent of his clinical deterioration, the longitudinal evaluation of MMSE, MoCA, and CDT was made, and he had a score on the MMSE of 24/30, 23/30, 22/30, and 10/30, MoCA of 21/30, 19/30, 18/30 and not completed, CDT of 4/4, 4/4, 4/4, and 3/4, respectively, at age 74, 75, 76, and 77 years (2 months before he died). Besides, modified Aphasia Battery of Chinese test for language evaluating was performed a second time at age 75, showing an extensive speech deficit, with spontaneous speech and writing, and sentence comprehension most seriously involved (Table 1).

**Figure 1:**
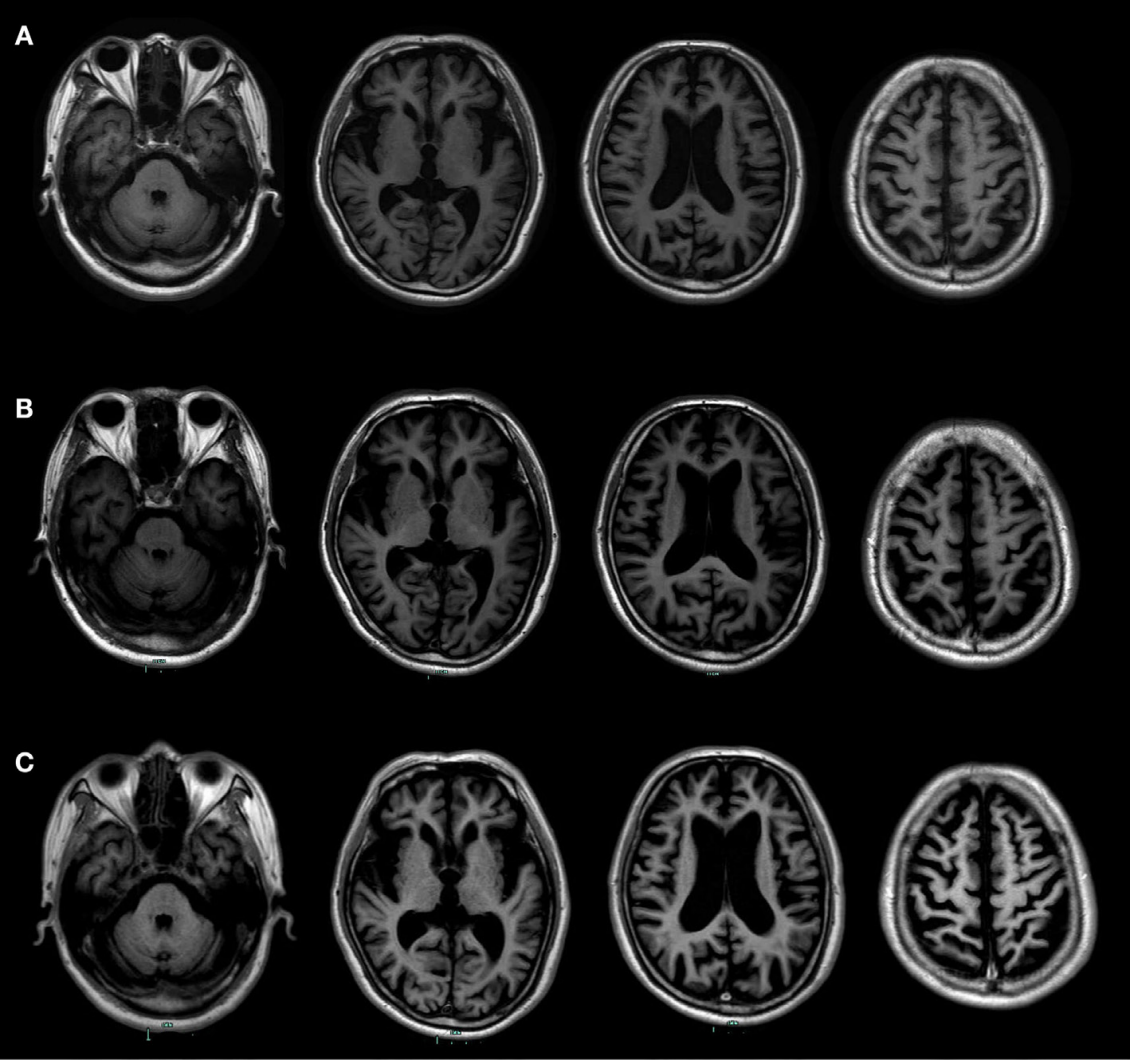
Longitudinal structural MRI scans showed progressive brain atrophy. **(A)** It was performed at the age of 73. **(B)** It was performed at the age of 74. **(C)** It was performed at the age of 76. Those follow-up MRI scans indicated asymmetric atrophy of the frontal, temporal, and parietal lobes that was more pronounced on the left side of the brain, especially on the left inferior frontal and insular.

**Figure 2:**
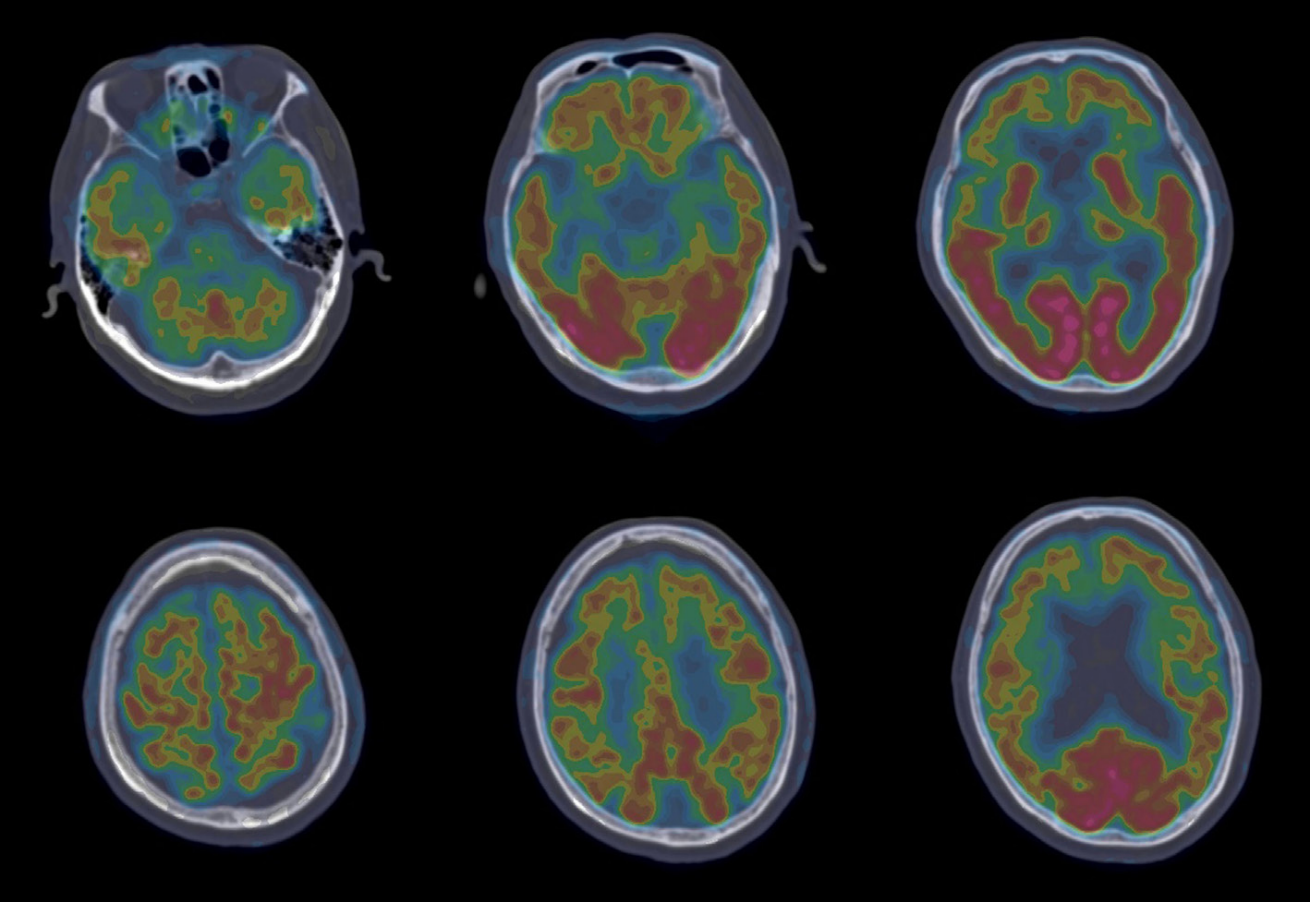
18F-FDG-PET revealed hypometabolism of the bilateral frontal, temporal, and parietal lobes that was more prominent on the frontal region.

Such determining tests as longitudinal structural MRI, functional imaging scanning, CSF biomarker screening, and gene sequencing were further performed. Longitudinal structural MRI showed gradually progressive structural atrophy and indicated asymmetric atrophy of the frontal, temporal, and parietal lobes which was much more pronounced on the left side of the brain (Figure 1). Furthermore, 18F-FDG-PET revealed hypometabolism of the bilateral frontal, temporal, and parietal lobes that was much greater on the frontal region (Figure 2), which was unanimous with regard to vital abnormalities revealed by his 99-Tc-SPECT scanning. CSF biomarker screening showed normal Aβ_42_ (708 pg/ml, normal >500 pg/ml) and total tau protein concentrations (t-tau, 285 pg/ml, normal <350 pg/ml), slightly elevated phosphorylated tau protein concentration (p-tau, 74 pg/ml, normal <50 pg/ml), and Aβ_42_/t-tau ratio of 2.5. Genomic DNA was obtained, and APP, PSEN1, PSN2, and APOE genes associated with AD, PGRN, MAPT, VCP, CHMP2B, TARDBP, and C9ORF72 genes associated with frontotemporal lobar degeneration were screened for mutations by sequencing. As a result, the sequence of APOE was e3/e4 and that of other genes were all normal. Besides, the next-generation sequencing of 81 established AD-related gene mutations turned out to be negative.

Therefore, this follow-up study with clinical identification, biofluid, and imaging biomarkers was performed to describe a 71-year-old man with gradually progressive loss of language and ultimately determine his probable presence of non-fluent/agrammatic variant of PPA (naPPA) with tau pathology, although a definitive diagnosis was not likely to be reached for lacking of microscopic assessment at autopsy. Meanwhile, this study showed the whole process of naPPA in detail.

## Discussion

This man presented speech disorder that began with speaking aloud with a slight lisp, which would be possibly ignored and create some confusion between the symptom of dysarthria and aphasia. Then on the basis of his longitudinal course, his speech disorder was characterized by non-fluent and effortful spontaneous speech, principal impairment of sentence repetition and comprehension, along with impairment of memory function. Therefore, his main diagnostic trait was aphasia and memory impairment.

Screening test was designed for contributing to our initial diagnosis of neurodegenerative disorder, as no evidence suggested such diseases as stroke, brain infection, neoplasm, trauma, toxic, and on account of a 6-year course of his progressive progression. Actually, his core deficit was exclusively aphasia; his memory function was substandard but still superior to language function, which could be totally explained by a gradual deterioration to extensive cognitive dysfunction and behavioral disorder and used to exclusively diagnose typical AD. With the disease going on, the disorder of personality and motor dysfunction was increasingly obvious, which was only second to language dysfunction; on the other hand, memory function progressed a little slowly. As a consequence, his clinical diagnosis was inclined to PPA. Certainly, determining tests were used to eliminate AD in this patient.

Primary progressive aphasia is a spectrum of selective language disorder, involving three common variants: semantic variant of PPA (SvPPA), logopenic variant of PPA (LvPPA), and naPPA (2). The typical characteristic linguistic change of SvPPA is impaired confrontation naming (7), that of LvPPA is impaired repetition of phrases and sentences and single-word retrieval (6), and that of naPPA is effortful speech with sound errors and grammatical simplification (8). In the current study, his function of naming was relatively spared, but phonological processing was destroyed, which was likely to exclude the clinical diagnosis of SvPPA. Meanwhile, one of his prominent presentations was poor sentence repetition, which was in line with the clinical feature of LvPPA. However, the most distinctive feature of this man was non-fluent and effortful speech, revealing deficit of sound, prosody, and grammar, which was most closely in accordance with the naPPA and not much supporting the LvPPA.

Different variants of PPA are associated with different cortical atrophies and pathological changes. The clinical imaging hallmark of SvPPA is in anterior and ventral temporal lobes (9), that of LvPPA is in the left posterior superior temporal and inferior parietal lobes (10), and that of naPPA involves cortical atrophy mainly in the left inferior frontal and insula (11). All those anatomical distributions of atrophy correspond to the functional imaging findings with SPECT and PET (12). Besides, evidence from most studies suggests that frontotemporal degeneration pathology changes result in naPPA (13). In addition, structural MRI, FDG-PET, and 99-Tc-SPECT of this patient were unanimous in vital abnormalities in the left inferior frontal and insular. CSF test revealed elevated p-tau protein and p-tau/Aβ1–42 ratio. Therefore, this patient met the clinical and imaging criteria for naPPA, and all the information of this man seems to be biased toward naPPA with tau pathology.

To our knowledge, few studies have systematically and longitudinally documented the characteristics of naPPA patients with Han language, which is a pictographic language. Evidence is converging that naPPA patients with Han language suffered a major damage of language expression in the early stage (14–16), manifesting as simple 1anguage, laborious speech, agrammatism, and wrong notes, with an average onset age of 61, and corresponding MRI changes mainly located in the left or bilateral frontotemporal area (17). We reported a similar linguistic and radiological feature, but with a relative complex disease course and an older-onset age. Besides, asymmetrical structural changes in the frontal and temporal lobes and corresponding language impairment have been reported in a Chinese PPA patient, indicating that his performance of Han language resembles Latin language. However, PPA phenotype is not explored in the study (18). Considering that overlapping clinical features have been existing in several neurodegenerative diseases, 18F-FDG PET images have been analyzed in a study of Chinese patients with naPPA, which has showed functional insulting in the left sylvian fissure, the posterior frontoinsular, and temporoparietal areas (16). Besides, genetic analysis has been performed in Chinese population, indicating that MAPT mutation presents in naPPA (19). The present study has referred to several genes associated with AD and frontotemporal lobar degeneration, but those genes including MAPT have been confirmed negative; however, his CSF biomarker screening has revealed possible tau pathology. As a whole, the present study has deepened our understanding of naPPA with Han language.

Overall, for a slowly progressively declined neurological disease, symptom changes with time, it is vital to recognize the core symptom and reach a symptomatic diagnosis at first and then conduct clinical localized and qualitative diagnosis. This case highlights the importance of considering a longitudinal course of a Chinese patient with speech disorder and comprehensively assessing his clinical characteristics with scale strategies, since a symptomatic diagnosis is vague. Second, the brain biopsy was irreplaceable but much limited, exploring the underlying pathology by CSF biomarker screening, functional imaging scanning, and gene sequencing which are helpful.

## Ethics Statement

Written informed consent was obtained from the guardian of the patient for publication of this case report and any accompanying images.

## Author Contributions

XL: drafting and revising the manuscript for content, analysis, and interpretation of data. FH: drafting and revising the manuscript for content. PL and ZC: collection, analysis, and interpretation of clinical data. GP: revising the manuscript for content, analysis, and interpretation of data, and accepting responsibility for the conduct of research and final approval.

## Conflict of Interest Statement

The authors declare that the research was conducted in the absence of any commercial or financial relationships that could be construed as a potential conflict of interest.

## Abbreviations

AD, Alzheimer’s disease; CDT, Clock-Drawing Test; LvPPA, logopenic variant of primary progressive aphasia; MMSE, Mini-Mental State Examination; MoCA, Montreal Cognitive Assessment; naPPA, non-fluent/agrammatic variant of primary progressive aphasia; PPA, primary progressive aphasia; SvPPA, semantic variant of PPA.
