# Supplementary material for: A Longitudinal Study of a Chinese Man Presenting with Non-Fluent/Agrammatic Variant of Primary Progressive Aphasia
Source: Front Neurol. 2018 Feb 16;9:75. doi: 10.3389/fneur.2018.00075 (PMC5820293; doi:10.3389/fneur.2018.00075)

In this supplementary file, regions of atrophy in this Chinese man were explored in comparison to a control group.

Methods

The patient's and five age and sex matched controls' anatomical images were calculated using voxel-based morphometry, based on VBM8 and SPM software, including normalizing to a template space, segmenting into grey and white matter, modulating to adjust for local volume changes introduced by the nonlinear normalization, and Gaussian smoothing (8 mm FWHM).

For statistical analysis, voxel-wise gray values of this patient were compared to those of the control group, and a 2-sample t-test (group 1: patient, group 2: controls) was adopted, while assuming the control data of normally distributed variables. Statistical thresholds were set at a corrected p value of $p < 0.05$, based on multiple comparisons using the Family Wise Error correction.

Results

Compared to the normal controls, this Chinese man showed significant decreased voxel-wise gray values of the frontal lobe, insular, caudate, temporal lobe and cerebellum, which were more prominent in frontal lobe and insula (Supplementary table 1 and Supplementary Fig.1).

Supplementary table 1. Brain regions showing significant differences between this Chinese man and normal controls

Brain regions	Cluster size (k)	MNI coordinate (X, Y, Z)			Peak T-value
Cerebellum_8_R	1682	35	-59	-29	-309
Cerebelum_Crus1_L	1531	-27	-69	-20	-481
Frontal_Inf_R/Insula_R/Temporal_Sup_R	5478	47	-15	12	-1455
Temporal_Inf_L/Frontal_Inf_L	1178	-44	-6	-36	-1291
Caudate_L/L Frontal sub-lobar	3047	-14	18	-5	-565
Precentral_L /insula_L	12601	-39	-35	20	-747
Thalamus_L	307	-11	-20	15	-694
Inf_Parietal_R	355	32	-45	45	-414

Cluster size was indicated by k = number of voxels in the particular cluster, and grey matter loss in this patient was compared to controls using a > 300 k voxels cut-off. L = left; R = right; inf = inferior; sup = superior.

Supplementary Fig. 1. Full view of significant differences of the voxel-wise gray values between this Chinese man and normal controls, using the BrainNet Viewer.

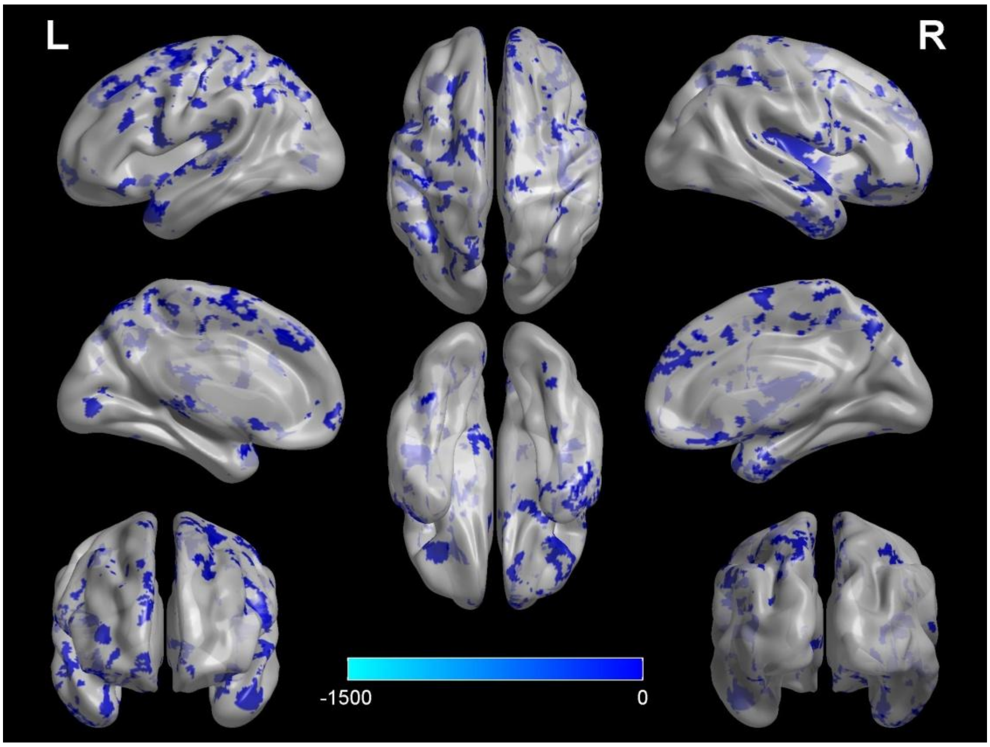

Supplement: Supplementary file 1 [file Presentation_1.PDF]
